# The protective effects of humanin in rats with experimental myocardial infarction: The role of asprosin and spexin

**DOI:** 10.1016/j.heliyon.2023.e18739

**Published:** 2023-07-26

**Authors:** Elif Onat, Nevin Kocaman, Serhat Hancer

**Affiliations:** a Department of Medical Pharmacology, Faculty of Medicine, Adiyaman University, Adiyaman, 02040, Turkey; b Department of Histology and Embryology, Faculty of Medicine, Firat University, Elazig, 23119, Turkey

**Keywords:** Myocardial infarction, Humanin, Asprosin, Spexin

## Abstract

**Objective:**

In this study, by applying humanin (HN) before myocardial infarction (MI) in rats, its protection in MI and the possible roles in asprosin and spexin were investigated.

**Materials and methods:**

The rats were divided into 7 groups each with 6 rats (group I (control), group II (HN 48th hour), group III (HN 7th day), group IV (MI 48th hour), group V (MI 7th day), group VI (MI + HN 48th hour), group VII (MI + HN 7th day). To create MI, 200 mg/kg isoproterenol (ISO) was administered subcutaneously to the rats. 2 mg/kg HN was given intraperitoneally (ip) to rats alone and before MI. Molecular parameters asprosin and spexin were examined by immunohistochemical in heart tissue. Biochemical parameters (aspartate aminotransferase (AST), lactate dehydrogenase (LDH), creatine kinase MB (CK-MB), Troponin I) were studied in serum by enzyme-linked immunosorbent assay (ELISA) method.

**Results:**

It was found in the study that the levels of spexin elevated especially towards the 7th day after MI and decreased more significantly towards the 7th day, after HN application before MI. Asprosin elevated significantly towards the 7th day after MI and decreased especially on the 7th day after HN application before MI. Also, serum AST, LDH, CK-MB, and Troponin I levels tended to decrease and a significant decrease was detected in congested veins in the heart tissue at the 48th hour of MI and erythrocyte extravasation, congested veins and necrotic muscle fibers at the 7th day of MI in rats given HN before MI.

**Conclusion:**

It was concluded that HN has a cardioprotective effect in MI and asprosin and spexin might mediate this effect.

## 1 Introduction

Acute Myocardial Infarction (AMI) is the most common reason of mortality among Coronary Vascular Diseases (CVD) in the world (15.9 million/year). Various new therapeutic strategies showed promise in preclinical studies in the last fifty years but failed in clinical trials [1]. New treatment modalities must be developed to reduce infarct size, improve survival and reduce hospitalization rates in CVD [2].

As an endogenous active peptide encoded by mitochondrial DNA, it was reported that humanin (HN) is associated with Acute Coronary Vascular Diseases (ACVD) [3] (1): serum HN level is negatively correlated with age [4]; (2) HN reduces H_2_O_2_-induced oxidative stress damage in myocardial cells and isolated myocardial mitochondria after promoting the expression of antioxidant defense system proteins and inhibiting the activity of complexes I and III of the electron transport chain [5]; (3) HN also reduces ROS production, protects endothelial cells from oxidative stress damage caused by abnormal glycolipid metabolism [6]; (4) and regulates Chaperone-Mediated Autophagy (CMA) after reducing ROS production because it regulates heat shock protein 90 (Hsp 90) and protects cardiomyocytes and fibroblasts from oxidative stress damage [7]; (5) HN upregulated antioxidant enzyme expression after reducing myocardial cell death and myocardial infarction (MI) area in an ischemia/reperfusion (I/R) injury model, protecting cardiac functions following MI [8]. It was also observed that the level of HN decreased in coronary heart disease patients and lactic acid level increased in comparison to normal people, which suggests that the protective effects of HN on the cardiovascular system are greater than its antioxidant effects [9]. HN is positively associated with coronary artery endothelial function and for this reason, it might be a novel target for coronary heart disease treatment [10].

A recent novel hormone called Asprosin, which has a protein structure, was discovered that regulates hepatic glucose secretion. This hormone is the *C*-terminal cleavage product of profibrillin-1 protein and is secreted from white adipose tissue and increases glucose and insulin secretion during fasting. It also stimulates the hypothalamic nutrition center and stimulates appetite and fat storage [11]. In some previous studies, it was reported that asprosin contributed to wound-healing with its protective effects on the myocardium. It was also observed that it significantly regulated left ventricular functions [12]. Asprosin is a new marker in angina pectoris and the severity of acute coronary syndrome [13].

Spexin is a new 14-amino acid neuropeptide (also “neuropeptide Q″) [14] which is encoded by the C12orf29 gene (on chromosome 12 of the human genome) [15] and is produced in human white adipose tissue [14]. It is expressed in various other tissues/organs (e.g., brain, heart, lung, liver, thyroid, adrenal, muscle, ovary, testis, pancreas, stomach and various parts of the gastrointestinal (GI) system [ [14,16,17]]. The role of spexin is not yet fully known, but the results of previous studies suggest a possible role in obesity regulation, energy homeostasis, appetite control, satiety, glucose, lipid metabolism, fatty acid intake, cardiovascular/renal function, endocrine homeostasis, reproduction and GI function [18].

It is known that various pharmacological agents have a protective effect due to their administration before/during ischemia or reperfusion. One of these, HN, is known to have a cardiac protective effect when applied before MI, but the effect of HN on asprosin and spexin is unknown. For this reason, this study aimed to investigate whether asprosin and spexin play a role in this effect of HN during myocardial infarction on rat with experimental procedure.

## 2 Materials and methods

### 2.1 Animals and experimental design

Animal Ethics Committee of Adıyaman University (Protocol No:2022/050) approved animal experiments. A total of male 42 Sprague-Dawley (8–10 weeks old) rats (200–250 g) provided by Adıyaman University Experimental Research Center were used and given ad libitum standard water and feed. The animals were divided into 7 groups (n:6); group I (control):No application was made to this group, group II (HN 48th hour): The rats in this group were administered 2 mg/kg HN alone (H6161, Sigma-Aldrich Corporation St. Louis, USA) [2] and blood samples were taken from the hearts in the rats at 48 h, group III (HN 7th day):The rats in this group were administered 2 mg/kg HN alone and blood samples were taken from the hearts in the rats on the 7th day, group IV (MI 48th hour): The rats in this group were administered subcutaneously 200 mg/kg ISO (Isoproterenol hydrochloride, I5627, Sigma-Aldrich Corporation St. Louis, USA) [19] to induce MI and blood samples were taken from the hearts in the rats at 48 h, group V (MI 7th day): The rats in this group were administered subcutaneously 200 mg/kg ISO to induce MI and blood samples were taken from the hearts in the rats on the 7th day, group VI (MI + HN 48th hour): HN (2 mg/kg) was administered to rats in this group before ISO administration at 10 min and the rats were administered subcutaneously 200 mg/kg ISO to induce MI and blood samples were taken from the hearts in the rats at 48 h, group VII (MI + HN 7th day): HN (2 mg/kg) was administered to rats in this group before ISO administration at 10 min and the rats in this group were administered subcutaneously 200 mg/kg ISO to induce MI and blood samples were taken from the hearts in the rats on the 7th day [19]. The rats were anesthetized with ip ketamine (75 mg/kg)+xylazine (10 mg/kg) before blood samples were taken from the hearts in the rats and the experiment was terminated. Then cardiac tissues were fixed in a 10% formaldehyde solution for histological studies and serum samples were stored at −80 °C for biochemical studies.

### 2.2 Histochemical examination

Cardiac tissues of animals were passed through routine histological follow-up series and embedded in paraffin blocks. Hematoxylin & Eosin, Masson Trichrome and Immunohistochemical stains were applied by taking 5 μm thick sections from these blocks.

### 2.3 Immunohistochemical examination

Immunohistochemical procedures were used as described earlier by Kocaman and Artas [20]. Immunohistochemistry (IHC) was performed using histological tissue microarray slides that were at 3 μm thick. The following antibodies were used: *Anti*-asprosin antibody (FNab09797; Fine Biotech Co., Wuhan, China) and Spexin primary antibody (A04088-1, boster biological technology, Pleasanton, CA, USA). Evaluated and photographed using the Zeiss Axio Scope. A1 microscope (Carl Zeiss Microscopy Gmb H 07745 Jena, Germany). As a result of immunohistochemical staining, a histoscore for Asprosin/Spexin was established.

Microscopic evaluation of the staining density was performed as: the areas with negative staining were given a value of 0, areas with <25% staining were given a value of 0.1, areas of 26–50% staining were given a value of 0.4, areas with 51–75% staining were given a value of 0.6, and areas of near homogeneous staining (76–100%) were given a value of 0.9. The final histoscore was then calculated using the formula: histoscore = distribution × intensity [20].

### 2.4 Serological analysis

An immunoassay analyzer (AQT90 FLEX; Radiometer, Copenhagen, Denmark) was used to analyze CK-MB and Troponin I assay levels. An Architect c8000 Chemistry System (Abbott Diagnostics) and commercial kits (Abbott Diagnostics) were used to analyze AST and LDH levels.

### 2.5 Statistical analysis

The SPSS 22 (IBM Corporation, USA) was employed in the analyses. The One-Way ANOVA Test was used and post-hoc multiple comparisons were made by using the Tukey HSD test. Kolmogorov Smirnov test was used for normal distribution test. Data are given as mean ± SD and p < 0.05 was considered statistically significant.

## 3 Results

### 3.1 ELISA findings

As a result of HN administration, serum AST, LDH, CK-MB, and Troponin I levels showed a slight increase in the 48th hours and 7th day compared to the control group (Table 1).

**Table 1:** ELISA findings.

Parameters	Control	HN 48th hour	HN 7th day	MI 48th hour	MI 7th day	MI + HN 48th hour	MI + HN 7th day
AST (U/L)	157.7 ± 50.33	171.4 ± 10.14	164 ± 6.36	481.4 ± 76.02 abc	213.7 ± 34.81 d	274.7 ± 40.46 abcd	164.4 ± 33.22 df
LDH (u/L)	563.4 ± 56.75	633.6 ± 21.17	620.4 ± 10.75	1013.2 ± 65.45 abc	1184.9 ± 59.94 abcd	671.6 ± 46.13 ade	684.6 ± 51.9 ade
CK-MB (U/L)	732.52 ± 73.45	794.77 ± 55.95	783.19 ± 17.52	1030.76 ± 55.4 abc	1800.4 ± 37.74 abcd	758.68 ± 19.08 de	885.12 ± 59.14 abcdef
Troponin I (μg/L)	0.0064 ± 0.004	0.0082 ± 0.003	0.007 ± 0.004	20.58 ± 4.04 abc	0.0099 ± 0.0003 d	11.66 ± 1.69 abcde	0.0076 ± 0.004 df

Error bars indicate SD. a. p < 0.05 in comparison to the control. b. p < 0.05 in comparison to HN 48th hour. c. p < 0.05 in comparison to HN 7th day. d. p < 0.05 in comparison to MI 48 h. e. p < 0.05 in comparison to MI 7th day. f. p < 0.05 in comparison to MI + HN 48th hours.

As a result of MI, LDH, and CK-MB levels elevated at the 48th hour and 7th day. Serum AST, Troponin I levels elevated at the 48th hour but elevated insignificantly on the 7th day of MI. LDH and CK-MB levels decreased at the 48th hour and 7th day in the MI groups given HN. AST and Troponin I levels were found to be lower at the 48th hour but decreased insignificantly on the 7th day in the MI groups given HN (Table 1).

### 3.2 Histochemical findings

**Fig. 1:**
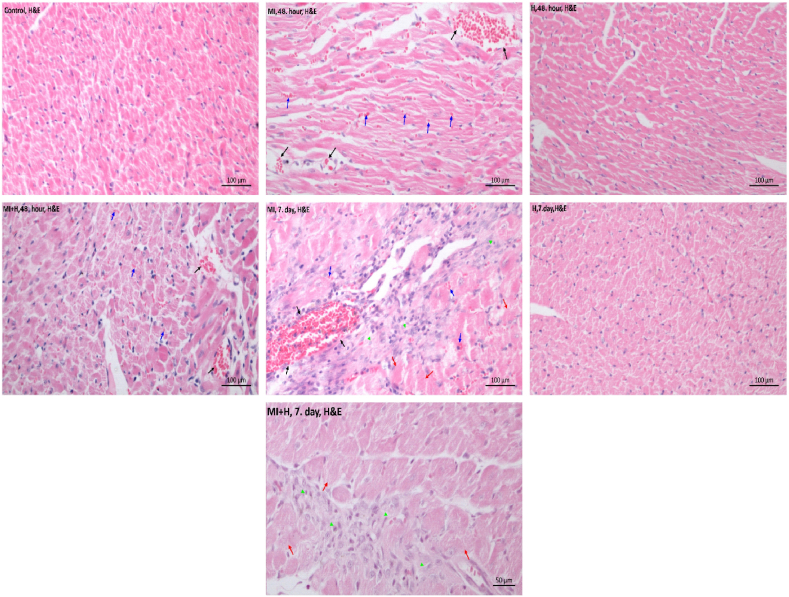
The histopathological examination of cardiac tissues (hematoxylin and eosin) over the entire time (control, HN 48th hour, HN 7th day, MI 48th hour, MI 7th day, MI + HN 48th hour, MI + HN 7th day) of observation.

**Fig. 2:**
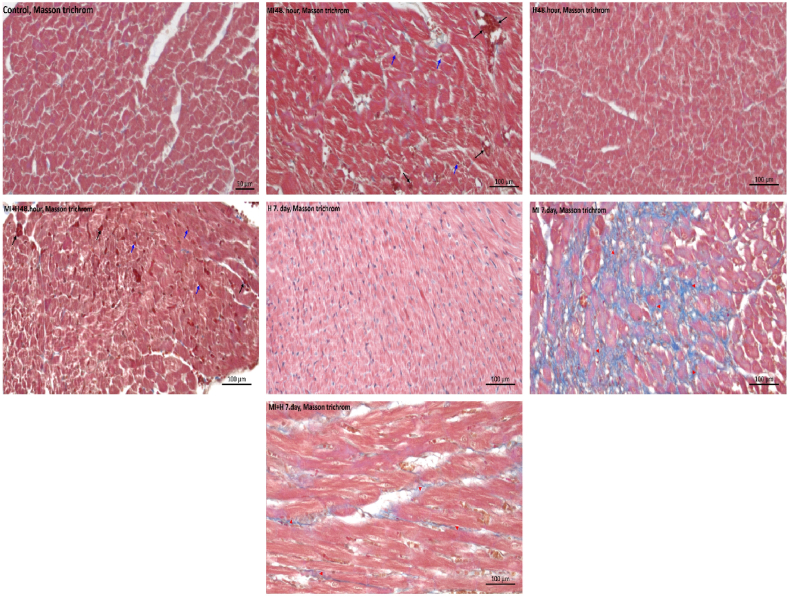
The histological findings of cardiac tissues for the entire time (Masson trichrome staining) (control, HN 48th hour, HN 7th day, MI 48th hour, MI 7th day, MI + HN 48th hour, MI + HN 7th day) of observation.

As a result of the examination of Hematoxylin-Eosin and Masson trichrome stained preparations of all groups under light microscopy, the control, HN 48th hour, and HN 7th day groups had normal appearance (Table 2) (Fig. 1, Fig. 2).

**Table 2:** Histopathologic findings of the cardiac tissues (hematoxylin and eosin).

Parameters	Control	HN 48th hour	HN 7th day	MI 48th hour	MI 7th day	MI + HN 48th hour	MI + HN 7th day
Necrotic Muscle Fibers	0	0	0	0.29 ± 0.49	6.86 ± 1.07 abcdf	0	4 ± 0.82 abcdef
Erythrocyte Extravasation	0.43 ± 0.53	0.57 ± 0.53	0.57 ± 0.53	3 ± 0.58 abc	4.71 ± 1.38 abcd	3.29 ± 0.76 abc	0.71 ± 0.49 def
Fibrosis	0	0	0	0	6.86 ± 1.07 abcdf	0	7.29 ± 1.11 abcdf
Congested Veins	0.57 ± 0.53	0.43 ± 0.53	0.29 ± 0.49	5.43 ± 1.13 abc	7.43 ± 0.53 abcd	3.86 ± 0.90 abcde	0.71 ± 0.49 def

Error bars indicate SD. a. p < 0.05 in comparison to the control. b. p < 0.05 in comparison to HN 48th hour. c. p < 0.05 in comparison to HN 7th day. d. p < 0.05 in comparison to MI 48th hour. e. p < 0.05 in comparison to MI 7th day. f. p < 0.05 in comparison to MI + HN 48th hour.

Erythrocyte extravasation and congested veins increase were observed at 48 h of MI compared to control, HN 48th hour, HN 7th day (p < 0.05). On the 7th day of MI significant increase in erythrocyte extravasation, congested veins, fibrosis, and necrotic muscle fibers were observed (p < 0.05) (Table 2) (Fig. 1, Fig. 2). Compared to the 48th hour of MI, there was a significant reduction in congested veins in the MI + HN 48th-hour group (p < 0.05). In comparison to the 7th day of MI, a significant reduction in erythrocyte extravasation, congested veins and necrotic muscle fibers were observed in the MI + HN 7th day group (p < 0.05) (Table 2) (Fig. 1, Fig. 2).

### 3.3 Immunohistochemical findings

**Fig. 3:**
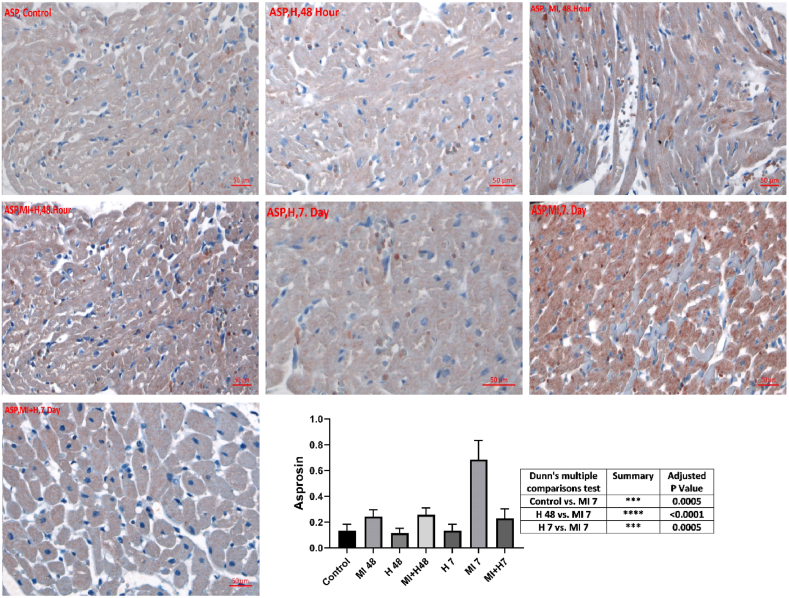
Immunohistochemical findings for asprosin in cardiac tissues (control, HN 48th hour, HN 7th day, MI 48th hour, MI 7th day, MI + HN 48th hour, MI + HN 7th day).

As a result of the observation of immunohistochemical staining for asprosin and spexin immunoreactivity in heart tissue under light microscopy;

Asprosin immunoreactivity was highly elevated on the 7th day of MI compared to control, HN 48th hour, HN 7th day, and MI 48th hour groups (p < 0.05). Asprosin immunoreactivity decreased in MI + HN 7th day group compared to the 7th day of MI (p < 0.05) (Table 3). Asprosin immunoreactivity histoscores for all seven groups are shown in Fig. 3.

**Table 3:** Immunohistochemical findings for asprosin in cardiac tissues.

Groups	Control	HN 48th hour	HN 7th day	MI 48th hour	MI 7th day	MI + HN 48th hour	MI + HN 7th day
Asprosin	0.12 ± 0.06	0.11 ± 0.04	0.12 ± 0.06	0.24 ± 0.05	0.69 ± 0.15 abcd	0.26 ± 0.05e	0.23 ± 0.08e

Error bars indicate SD. a. p < 0.05 in comparison to the control b. P < 0.05 in comparison to HN 48th hour. c. p < 0.05 in comparison to HN 7th day. d. p < 0.05 in comparison to MI 48th hour. e. p < 0.05 in comparison to MI 7th day.

**Fig. 4:**
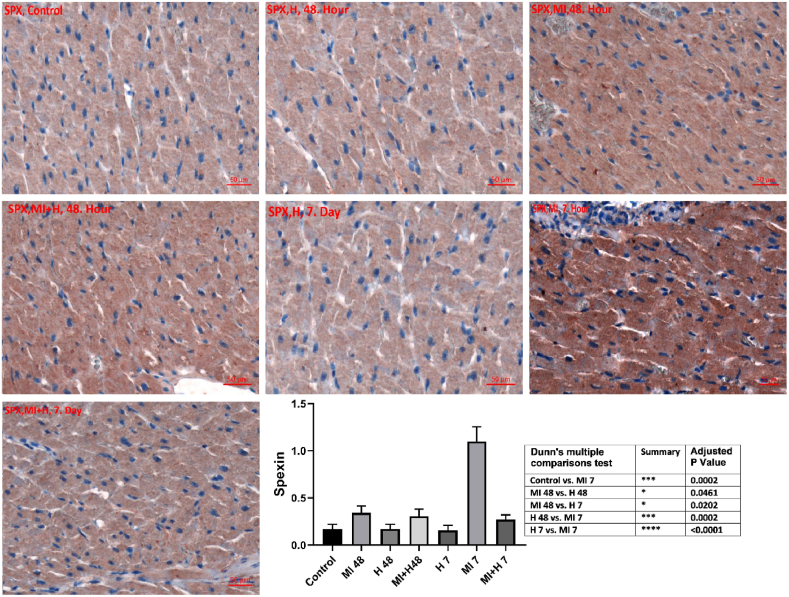
Immunohistochemical findings for spexin in cardiac tissues (control, HN 48th hour, HN 7th day, MI 48th hour, MI 7th day, MI + HN 48th hour, MI + HN 7th day).

Spexin immunoreactivity was seen as highly elevated on the 7th day of MI compared to control, HN 48th hour, HN 7th day, and MI 48th hour groups (p < 0.05). Spexin immunoreactivity decreased in MI + HN 7th day group according to the 7th day of MI (p < 0.05) (Table 4). Spexin immunoreactivity histoscores for all seven groups are shown in Fig. 4.

**Table 4:** Immunohistochemical findings for spexin in cardiac tissues.

Groups	Control	HN 48th hour	HN 7th day	MI 48th hour	MI 7th day	MI + HN 48th hour	MI + HN 7th day
Spexin	0.17 ± 0.05	0.17 ± 0.05	0.16 ± 0.05	0.34 ± 0.07	1.19 ± 0.27 abcd	0.31 ± 0.07 e	0.27 ± 0.05 e

Error bars indicate SD. a. p < 0.05 in comparison to control b. P < 0.05 in comparison to HN 48th hour. c. p < 0.05 in comparison to HN 7th day. d. p < 0.05 in comparison to MI 48th hour. e. p < 0.05 in comparison to MI 7th day.

## 4 Discussion

Ischemic heart disease is a serious threat to human life and health and there is an urgent need for novel therapeutic modalities for the treatment of MI. The effects of cellular and molecular mechanisms on the pathological processes of MI were taken into account in the past years, as well as the therapeutic strategies and treatment procedures developed. Many of the current strategies in MI treatment include promising clinical applications in the recovery of MI (e.g., pharmacotherapy, gene therapy, protein therapy, cell therapy, and exosome therapy) [21]. Although the protective effect of HN, an endogenous active peptide discovered as a result of these studies, on myocardial damage has been demonstrated by in vivo and in vitro studies, the exact mechanisms by which HN provides this protective effect are not known. In this study, in which the protection of HN in myocardial damage and the role of asprosin and spexin in this effect was investigated, it was shown for the first time that asprosin and spexin might be involved in this mechanism of action as supported by biochemical and histological findings that HN has a cardioprotective effect with its application before MI.

The HN analog reduced the size of the infarct in a dose-dependent manner in a mouse model with myocardial ischemia/reperfusion (MI/R) injury [22] in a previous study. Also, HN (2 mg/kg) administration improved left ventricular (LV) function 1 week after the MI/R injury [23]. In line with these findings, we confirmed the protective effects of HN and the toxic effect of ISO with biochemical and histological examinations. Also, an increase in plasma levels of troponin-I, LDH, AST, and CK-MB activities occurred with the application of ISO and serious damage and inflammation such as erythrocyte extravasation, congested vessel and necrotic muscle fiber increase in myocardial cells and deterioration in heart function were detected. Serious damage and inflammation such as erythrocyte extravasation, congested vessel, and necrotic muscle fiber increase occurred in the myocardial cell and resulted in deterioration in heart function. Interestingly, in the HN-treated groups, HN pretreatment showed a significant reduction in the amounts of all these cardiac markers. Current findings suggest that HN has a cardioprotective role by maintaining myocardial membrane integrity and thus restricting the leakage of these enzymes into the bloodstream.

Asprosin was reported as a promising cardioprotective agent by Zhang et al. [22] thinking that it regulates the function and survival of mesenchymal stromal cells (MSC) and that their use in the treatment of MI has a positive effect on the efficacy of MSCs [22]. Asprosin pretreatment in MI increased the targeting of MSCs to the lesion site. Also, intracardiac MSC pre-incubated with asprosin administration improved cardiac ejection with infarction and decreased cardiac fibrosis and reduced free radical-induced MSC damage and apoptosis after activating the ERK1/2 and PI3K/AKT pathways, upregulating SOD-2 antioxidative enzyme expression, and inhibiting further free radical production. Asprosin's cardioprotective effects were demonstrated by Wen et al. [23] in a cellular model showing that asprosin protects cardiomyocytes via hypoxia-induced apoptosis. But, clinical studies showed that dilated cardiomyopathy (DCM) patients with elevated asprosin levels had lower risks of negative clinical outcomes than patients with lower asprosin levels (<210 ng/mL). Chen et al. [24] showed that asprosin has protective effects against the damage to cardiac microvascular endothelial cells caused by elevated glucose concentration. The hypothalamic paraventricular nucleus (PVN) is an important central integrative area in the regulation of cardiovascular activity and is the main source of excitatory propulsion for sympathetic outflow to the spinal cord with both direct/indirect projection [3]. In hypertension and chronic heart failure, excessive sympathetic activation is associated with alterations in molecular signaling in PVN [3,4]. A study conducted on rats reported that asprosin increased sympathetic outflow, blood pressure and heart rate in PVN over cAMP-PKA signal-mediated NADPH oxidase activation and superoxide production and activated the cAMP-PKA pathway to cause sympathetic activation in this study. It is considered that asprosin's effects in PVN might be mediated by OR4M1 or OLFR734 receptors. Not all asprosin receptors in the PVN were identified to modulate sympathetic outflow since specific antagonists of the OR4M1 or OLFR734 receptors are not yet available. However, the possibility that long-term asprosin administration may increase the expressions of antioxidant enzymes in the PVN via its direct or secondary mechanism [25]. In our study, in which we created an MI model by inducing with ISO in accordance with these studies, we found that asprosin levels elevated especially towards the 7th day due to the activation of the sympathetic system. The decrease in asprosin levels on the 7th day after HN administration suggests that asprosin might play a role in the protective mechanism of HN through cAMP-PKA signal-mediated NADPH oxidase activation. However, it can be considered that asprosin might be associated with other pathways (Keap 1/Nrf 2, autophagy, JNK/p38 MAPK, AMPK, PI3K/Akt and JAK2/STAT3) effective in the protection of HN on the cardiovascular system. Although more studies are required to elucidate the mechanism of action of a newly discovered hormone, asprosin, in line with the findings of the current study, it is possible to conclude that asprosin might be a marker in MI and might mediate the protective effects of HN in MI.

In one of the studies, spexin-injected (i.c.v.) rats showed increased mean arterial pressure and decreased heart and urine flow rates, which suggests that central speciation plays roles in modulating cardiovascular/renal activities [26]. It was shown that spexin mRNA levels of the carotid body were higher in rats exposed to hyperoxia than in the group exposed to normoxia, which suggests that spexin in the carotid body might be a regulator of hyperoxia-induced plasticity [27]. In other words, it can be thought that spexin might have a defensive effect by increasing under stress and the increase in spexin level towards the 7th day especially due to MI in our study supports this information. On the other hand, in rats treated with HN before MI, the level of spexin decreased significantly on the 7th day. This finding can be evaluated that HN exerts its protective effect by reducing spexin levels. It is known that HN protects endothelial cells against oxidative stress damage caused by abnormal glycolipid metabolism by reducing ROS production. Spexin, on the other hand, is a peptide associated with glucose and lipid metabolism and might also act on ROS and play a role in the protective mechanism of HN. However, there are not enough studies in the literature on the subject.

The most important limitations of this study were that the findings were not supported by Western blot analysis and imaging methods such as ECG/ECO. Since our study was terminated on the 7th day of MI, we do not know whether HN showed more protective effects after the 7th day of MI and whether it reduced asprosin and spexin levels to their normal limits. Further studies explaining the relationship of HN with asprosin and spexin will better elucidate the molecular mechanisms of our study. More studies are needed to evaluate the clinical use of HN in patients with coronary heart disease. However, other signaling elements involved in HN-induced myocardial protective effects need to be identified.

In conclusion, in this study, it has been shown that asprosin and spexin may play a role in this effect of HN in MI, as biochemically and histopathologically supported that HN may have a protective effect in MI. Thus, a contribution has been made to the literature on the treatment of MI and its mechanisms of action.

## Author contribution statement

Elif Onat: Conceived and designed the experiments; Performed the experiments; Analyzed and interpreted the data; Contributed reagents, materials, analysis tools or data; Wrote the paper.

Nevin Kocaman: Analyzed and interpreted the data.

Serhat Hançer: Contributed reagents, materials, analysis tools or data.

## Data availability statement

Data included in article/supp. Material/referenced in article.

## Declaration of competing interest

The authors declare that they have no known competing financial interests or personal relationships that could have appeared to influence the work reported in this paper.
